# Use of traditional Chinese medicine as an adjunctive treatment for COVID-19: A systematic review and meta-analysis

**DOI:** 10.1097/MD.0000000000026641

**Published:** 2021-07-30

**Authors:** Feng Li, Yongqing Jiang, Bei Yue, Lili Luan

**Affiliations:** a Department of Infectious Diseases, Zhongshan Hospital of Xiamen University, Xiamen, Fujian Province, People's Republic of China; b Main Examination Room, Xiamen Huli Guoyu Clinic, Co., Ltd., Xiamen, Fujian Province, People's Republic of China; c Department of Endocrinology, The 73^rd^ Military Hospital of PLA/Cheng Gong Hospital Affiliated to Xiamen University, Xiamen, Fujian Province, People's Republic of China.

**Keywords:** adjunctive treatment, COVID-19, meta-analysis, traditional Chinese medicine

## Abstract

**Background:**

This review aims to evaluate the supportive effects of frequently used traditional Chinese medicine (TCM) for the treatment of coronavirus disease 2019 (COVID-19).

**Methods:**

Five databases were searched through July 7, 2020. Randomized controlled trials investigating the efficacy of TCM for use in the treatment of COVID-19 were included. Newcastle–Ottawa Scale (NOS) and modified Jadad score were used for the evaluation of the methodological quality of the included studies. Weighted mean difference, odds ratio (OR), and 95% confidence interval (95% CI) were calculated for pooling out results. Data were extracted for conducting a meta-analysis using STATA version 12.0.

**Results:**

Eight studies with a total of 750 patients were included in this meta-analysis. All included trial groups involved treatment with TCM and Western medicine, while the control groups were treated only with Western medicine. The intervention therapy significantly improved the overall effective rate (n = 346, OR = 2.5, 95% CIs = 1.46–4.29), fever symptom disappearance rate (n = 436; OR = 3.6; 95% CIs = 2.13–6.08), fatigue symptom disappearance rate (n = 436; OR = 3.04; 95% CIs = 1.76–5.26), cough symptom disappearance rate (n = 436; OR = 2.91; 95% CIs = 1.36–6.19), and sputum production reduction (n = 436; OR = 5.51; 95% CIs = 1.94–15.64). Based on the Newcastle–Ottawa Scale assessment, 6 studies received a score of 4, and 1 study achieved a score of 5. One study was assessed using the modified Jadad score, achieving a score of 6.

**Conclusions:**

The integration of TCM with Western medicine has significantly improved the treatment for COVID-19 patients compared to Western medicine treatment alone. Combined therapy using TCM and Western medicine revealed the potential adjunctive role of TCM in treating COVID-19. However, high-quality clinical studies are still required to further evaluate the efficacy and safety of TCM in the treatment of COVID-19.

## 1 Introduction

Coronavirus disease 2019 (COVID-19), which is caused by severe acute respiratory syndrome coronavirus 2 (SARS-CoV-2), emerged at the beginning of December, 2019 in China and has rapidly spread to many countries. Although the outbreak started from wild animals to human transmission, it was soon confirmed that the human-to-human transmission pathway also existed. Moreover, considering that SARS-CoV-2 spreads through close contact, infected droplet, or fomite, the raid spread of COVID-19 is not surprising, thus ravaging global public health.

Until March, 2021, COVID-19 has already caused more than a million deaths and is probably the severest disease in a century as reported by the World Health Organization (WHO).^[1]^ Patients with COVID-19 show quite wild clinical symptoms, including asymptomatic infection, fever, fatigue, dry cough, upper airway congestion, sputum production, shortness of breath, and severe viral pneumonia along with respiratory failure.^[2,3]^ The outbreak has been a big challenge to humans because even the most robust health systems have been overwhelmed and unable to adequately provide essential health services and care. Thus far, no licensed medicines for indications have been approved by the National Medicine Regulatory Authority.

Therefore, people have tried to use conventional therapeutics against COVID-19. Because SARS-CoV-2 shares genetic similarity to SARS-CoV,^[4]^ conventional drugs used for treating SARS could be an option. For example, lopinavir/ritonavir, which is a US Food and Drug Administration-approved protease inhibitor for treating human immunodeficiency viruses, was reported in the early-stage treatment of patients with SARS.^[5]^ Ribavirin, in combination with corticosteroids, was also widely used in SARS based on broad-spectrum antiviral activity.^[5]^ Both drugs were considered.^[6,7]^ Moreover, corticosteroid use in the treatment of COVID-19 was widely reported because corticosteroids were previously used to treat H1N1 viral pneumonia.^[8–10]^ Another drug, remdesivir, used as an antiviral agent, was reported in the treatment of SARS-CoV-2 infection and achieved good results both in vitro and in vivo.^[11,12]^ Chloroquine and hydroxychloroquine, known for the prevention and treatment of malaria and chronic inflammatory diseases, were also reported for use in the treatment of COVID-19.^[13,14]^ Both agents are relatively well tolerated, as demonstrated; however, as a complication, chloroquine and hydroxychloroquine can cause rare and severe adverse effects (<10%), including QT interval prolongation and hypoglycemia.

In China, traditional Chinese medicine (TCM) has been used as a therapy for infectious diseases including SARS, influenza H1N1, avian influenza, and malaria.^[15,16]^ The National Health Commission of China has declared that herbal medicine combined with Western medicine can be used for the treatment of COVID-19. Clinical evidence has shown favorable effects for the use of TCM.^[17]^ The results showed that TCM has played an indispensable role and no patients had progression from mild to critical disease, and no nurses and doctors were infected.^[18]^

Several systematic reviews or meta-analyses that included evidence from case reports, case series, and observational studies have also been conducted to study the effectiveness of herbal medicine in the treatment of COVID-19.^[19,20]^ However, in the present study, we focused only on the recently and most frequently used TCM in the therapy of COVID-19 patients, which may be helpful for a better understanding of the use of Chinese medicine in the therapy process. From pooling out results, the intervention (TCM plus Western medicine therapy) groups showed significant improvement with regard to the total effective rate as well as the lung computed tomography (CT) screening rate compared to the control groups (Western medicine therapy alone). In addition, regarding the main clinical symptoms (including fever, fatigue, cough, and sputum production) in COVID-19 patients, a significant reduction was reported in the intervention groups compared to the control groups. Finally, the results indicated that TCM could be considered as an adjunctive therapeutic option in the management of COVID-19.

## 2 Methods

### 2.1 Search strategy

This meta-analysis was conducted in accordance with the Preferred Reporting Items for Systematic Reviews and Meta-Analyses guidelines.^[21]^ Two authors (FL and YJ) conducted a systematic literature search in the following electronic bibliographic databases: (English database) PubMed and Embase; (Chinese database) Chinese National Knowledge Infrastructure Database, Chinese Science and Technique Journals Database, and the Wanfang Database.

All enclosed databases will be searched from the available date of inception to July 2020. The search strategy included the following terms: (“2019-nCoV” or “COVID-19” or “SARS-CoV-2”) and (“therapy”) and (“Chinese traditional medicine” or “Chinese medicine” or “Chinese herbal medicine” or “Chinese patent medicine”). Any indexed terms equivalent to “COVID-2019” and “Chinese traditional medicine” were also searched to extend the search coverage. There were no restrictions concerning the language or publication type.

### 2.2 Eligibility criteria

#### 2.2.1 Types of studies

Studies that included TCM as a treatment approach for COVID-19 were recruited in this meta-analysis if they were randomized controlled trials (RCTs).

#### 2.2.2 Types of participants

Patients were included if they met the following criteria: (1) diagnosed as COVID-19 positive, regardless of the sex, age, and ethnicity; (2) presented reverse transcription-polymerase chain reaction nucleic acid test-positive results; (3) with no other life-threatening diseases; and (4) without recent use of herbal medicine.

#### 2.2.3 Types of intervention groups

All types of oral administration and injections of TCM treatment or Chinese patent medicine combined with conventional treatment were retrieved. Only Chinese medicine treatment, herbal injections, or 2 or more different types of Chinese medicine were excluded. There were no limitations on the intake dosage, composition of Chinese medicine, or duration of treatment. Control groups that received only conventional methods for the treatment of COVID-19 were included. Conventional methods included simple nutrition, symptomatic, antiviral, and antibacterial treatment.

#### 2.2.4 Outcome measures

The primary outcome measures were total effective rate, the effective rate of lung CT, and aggravation or hospitalization. The effective rate was defined as the number of patients whose total symptom score was reduced by greater than or equal to 30% after treatment.

The secondary outcome measures were clinical symptoms (fever, fatigue, cough, and sputum disappeared) and clinical symptom duration time. The eligibility of the relevant studies and the data were independently assessed by the review author (FL). Subsequently, 2 review authors (FL and BY) conducted the data extraction using a standard form according to the inclusion and exclusion criteria. The information extracted in detail is as follows: title of the study, authors’ name, publication time, therapeutic schedule in the intervention groups (including the trial groups and the control groups), involved patient age and sex, body temperature, duration time, outcome measures, and study results. A third review author (LL) could be consulted if disagreements were identified. The corresponding author could be contacted through email if any detailed information on outcome measures was missing.

### 2.3 Methodological quality assessment

Quality assessment of the included studies was conducted according to modified Jadad scores and the Newcastle–Ottawa Scale (NOS). Randomized controlled trials used Jadad scale scores, and non-RCTs used NOS scale scores.

### 2.4 Statistical analysis

STATA version 12 software (StataCorp, College Station, TX) was utilized to conduct data analysis, which was extracted from original studies. Odds ratios (ORs) with 95% confidence intervals (CIs) were used for categorical variables. Continuous variables were summarized with a weighted mean difference (WMD) with 95% CIs. We used the *I*-squared statistic, a quantitative measure describing inconsistency across studies measured from 0% to 100%, to assess the between-study heterogeneity in the presented meta-analysis. For *I*-squared <50%, a fixed-effects model was applied. The Funnel test was used to evaluate publication bias. There was a significant difference when *P* < .05.

## 3 Results

### 3.1 Literature search

**Figure 1:**
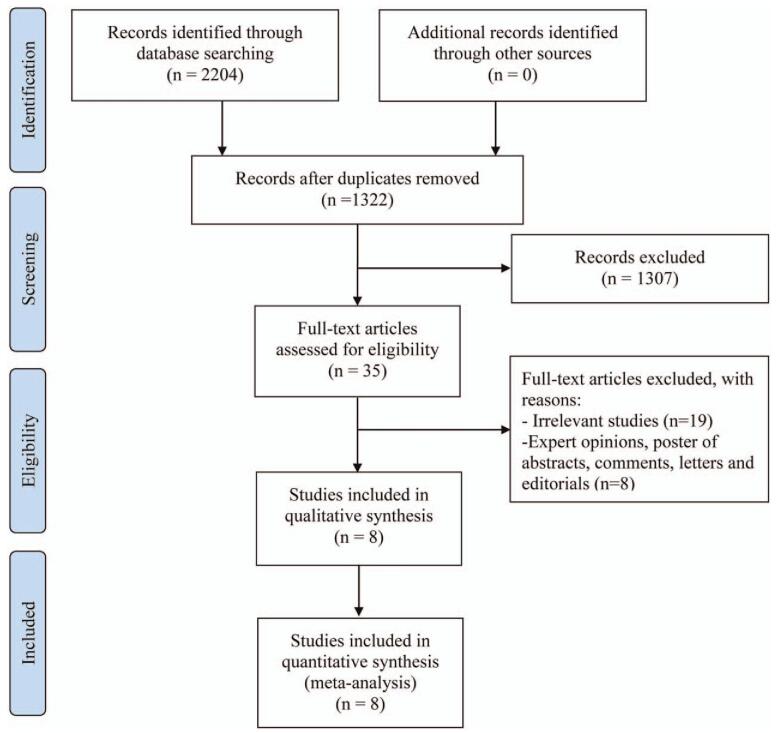
Flow chart depicting the literature search. Finally, only 8 studies were included in this review after series of quality assessments.

The database search identified 2204 studies, as shown in Figure 1. A total of 1322 studies were retrieved by screening the study titles and abstracts after removing duplicates, and another 1307 articles were excluded because they were not RCTs. Only 35 RCTs were assessed for eligibility, and then the full article of the studies was retrieved. On the basis of the predefined inclusion and exclusion criteria, we further excluded 27 studies as follows: 19 RCTs were excluded because of irrelevance. Eight studies were further excluded because they were expert opinions, posters of abstracts, comments, letters, and editorials. Finally, 8 studies were included in this review.

### 3.2 Study characteristics

All 8 RCTs were conducted in Mainland, China in 2020.^[22–29]^ The sample size was 750 in total. Except for 1 trial that did not report the sex composition, male and female patients accounted for 365 (56%) and 286 (44%), respectively. The participants’ age ranged from 33 to 75 years. Moreover, all trial groups included in this meta-analysis were applied with TCM (including Shu-feng-jie-du, Lian-hua-qing-wen, Jin-hua-qing-gan, and Xue-bi-jing) plus Western medicine (eg, antiviral drugs, antibacterial drugs, interferon, and arbidol) for COVID-19 treatment. The control groups were treated only with Western medicine. Applied administration included oral and intravenous injections. In addition, the dosage of Chinese medicine ranged from 2to 10 g, as shown in Table 1. The Xue-bi-jing injection dosage was 50 mL. Drugs were administered twice or thrice a day. All characteristics are presented in Table 1.

**Table 1:** Basic information and quality evaluation of the included studies.

		Trial group				Control group						
Author	Publishing time	(n)	Sex (M/F)	Age (yrs)	Treatment	(n)	Sex (M/F)	Age (yrs)	Treatment	Temperature (°C)	Course (days)	NOS score
Chen et al^[25]^	2020.6	34	14/20	65.1 ± 10.6	Conventional methods + Shufengjiedu 2g, P.O., tid	34	15/19	64.4 ± 10.3	Conventional methods	38.8 ± 0.75	7	4
Cheng et al^[22]^	2020.3	51	26/25	55.5 ± 12.3	Conventional methods + Lianhuaqingwen 6g, P.O., tid	51	27/24	55.8 ± 11.6	Conventional methods	38.3 ± 0.64	7	4
Duan et al^[26]^	2020.3	82	39/43	52.0 ± 13.9	Conventional methods + Jinhuaqinggan 10g P.O., tid	41	23/18	50.3 ± 13.2	Conventional methods	NR	5	6^∗^
Lyu et al^[27]^	2020.4	63	NR	59.0 ± 16.6	Conventional methods + Lianhuaqingwen 6g, P.O., tid	38	NR	60.2 ± 17.0	Conventional methods	38.0 ± 0.65	10	4
Qu et al^[29]^	2020.3	40	25/15	40.7 ± 8.2	Conventional methods + Shufengjiedu 2g, P.O., tid	30	16/14	39.8 ± 6.4	Conventional methods	38.7 ± 0.65	10	4
Xiao et al^[23]^	2020.3	100	64/36	60.90 ± 8.7	Conventional methods + Shufengjiedu 2g, P.O., tid	100	66/34	62.20 ± 7.50	Conventional methods	NR	14	5
Yao et al^[28]^	2020.2	21	16/5	57.1 ± 14.0	Conventional methods + Lianhuaqingwen 6g, P.O., tid	21	12/9	62.4 ± 12.3	Conventional methods	38.5 ± 0.65	NR	4
Zhang et al^[24]^	2020.4	22	10/12	49.1 ± 14.2	Conventional methods + Xuebijing 50 mL, Iv.gtt., bid	22	12/10	46.0 ± 14.7	Conventional methods	NR	7	4

### 3.3 Assessment of methodological quality

As shown in Table 1, the included study methodological quality was evaluated based on the NOS or the modified Jadad score. Based on the NOS, there were 6 studies that had a score of 4, and 1 study achieved a score of 5. There was only 1 study assessed on the basis of the modified Jadad score, with a score of 6.

### 3.4 Primary outcome measures

#### 3.4.1 Total effective rate

**Figure 2:**
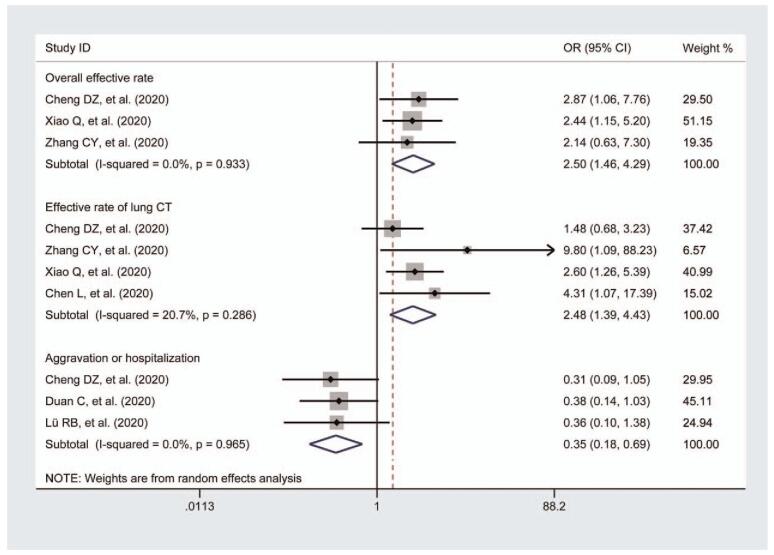
Meta-analysis for the comparison of total effective rate, the effective rate of lung CT, aggravation, or hospitalization between the 2 groups. In primary outcome measurements, pooling of data revealed that regarding the total effective rate, the effective rate of lung CT, and the aggravation or hospitalization of the treatment of COVID-19, the intervention of TCM and Western medicine showed a significantly better effect compared to Western medicine treatment. No significant heterogeneity analysis of each subgroup was observed. The fixed-effects model was used for statistical analysis. COVID-19 = coronavirus disease 2019, CT = computed tomography, TCM = traditional Chinese medicine.

Three studies^[22–24]^ assessed the overall effective rate of the treatment of COVID-19. Pooling of data revealed that patients treated with combined TCM and Western medicine showed a significantly better effect in terms of the total effective rate (n = 346, OR = 2.5, 95% CIs = 1.46–4.29) (Fig. 2). A fixed-effects model was used for the statistical analysis. No significant heterogeneity was found (*I*-squared = 0.0%, *P* for heterogeneity = .933).

#### 3.4.2 Effective rate of lung CT

A total of 4 studies^[22–25]^ provided data regarding the effective rate of lung CT, to observe the changes after the intervention of Chinese medicines plus Western medicine in COVID-19 patients. The meta-analysis showed a significant effect of combined therapy on symptom disappearance in screened patients (n = 414, OR = 2.48, 95% CI = 1.39–4.43) (Fig. 2). A fixed-effects model was adopted and revealed no clear heterogeneity among the studies (*I*-squared = 20.7%, *P* for heterogeneity = .286).

#### 3.4.3 Aggravation or hospitalization

Three studies^[22,26,27]^ reported on aggravation or hospitalization after the intervention. There were 196 patients in the trial group and 130 patients in the Western medicine group. No severe or aggravation patients were found (n = 326; OR = 0.35; 95% CI = 0.18–0.69) (Fig. 2). No significant heterogeneity analysis (*I*-squared = 0.0%, *P* for heterogeneity = .933) was observed. Therefore, a fixed-effects model was used for statistical analysis.

### 3.5 Secondary outcome measures

#### 3.5.1 Fever disappearance

**Figure 3:**
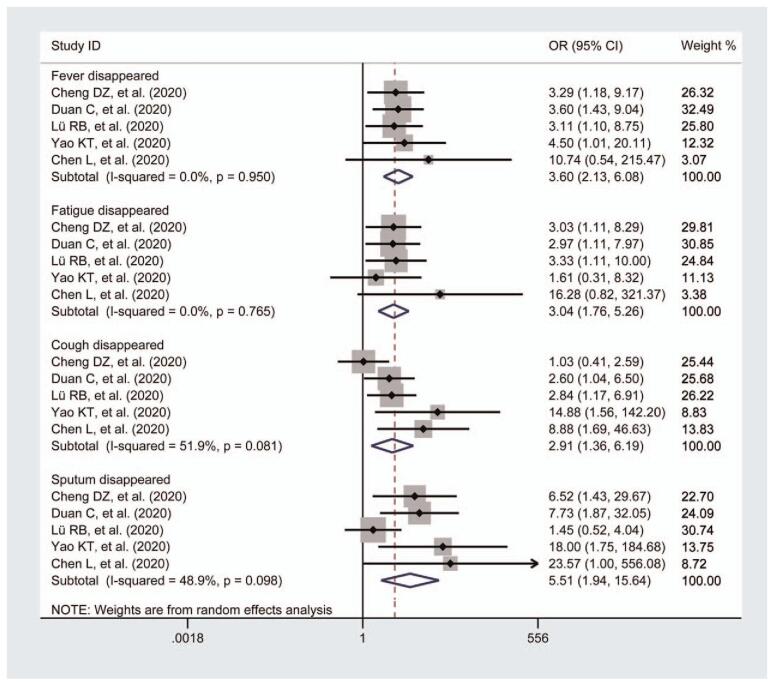
Meta-analysis for the comparison of the disappearance of the main symptoms including fever, fatigue, cough, and sputum between the 2 groups. As the secondary outcome measurement, fever disappearance, fatigue disappearance, cough disappearance, and sputum disappearance were reported. The combined therapy groups showed a significantly greater effect in COVID-19 patients. Only a low heterogeneity was observed in the cough disappearance subgroup. COVID-19 = coronavirus disease 2019

Fever disappearance was reported in 5 studies.^[22,25–28]^ There was only 1 study that did not report the body temperature change.^[26]^ In the 5 RCTs, 251 patients were in the intervention group and 185 in the control group. All of the studies revealed significant differences between trial groups and control groups (n = 436; OR = 3.6; 95% CI = 2.13–6.08) (Fig. 3). No heterogeneity was observed (*I*-squared = 0.0%, *P* for heterogeneity = .950).

#### 3.5.2 Fatigue disappearance

Five studies^[22,25–28]^ showed fatigue disappearance in the patients after the intervention. In comparison to Western medicine groups, the combined therapy groups showed a significantly greater effect on the disappearance of fatigue in COVID-19 patients (n = 436; OR = 3.04; 95% CIs = 1.76–5.26) (Fig. 3). A fixed-effect model was used for statistical analysis in terms of no heterogeneity being observed (*I*-squared = 0.0%, *P* for heterogeneity = .765).

#### 3.5.3 Cough disappearance

Cough disappearance time was reported in 5 studies.^[22,25–28]^ In the field of the number of cough reduction cases, there were 251 patients in the intervention group and 185 in the control group. The meta-analysis revealed a significant improvement in the cough disappearance cases when intervened with TCM (n = 436; OR = 2.91; 95% CI = 1.36–6.19) (Fig. 3). A low heterogeneity (*I*-squared = 51.9%, *P* for heterogeneity = .081) was observed in the pooled outcomes.

#### 3.5.4 Sputum disappearance

Five studies assessed sputum reduction^[22,25–28]^ and reported that sputum from patients treated with a combination of Chinese medicines and Western medicine was significantly reduced (n = 436; OR = 5.51; 95% CI = 1.94–15.64) (Fig. 3). There was no heterogeneity observed (*I*-squared = 48.9%, *P*for heterogeneity = .098); a fixed-effects model was used for statistical analysis as before.

### 3.6 Duration of symptoms

#### 3.6.1 Fever duration

**Figure 4:**
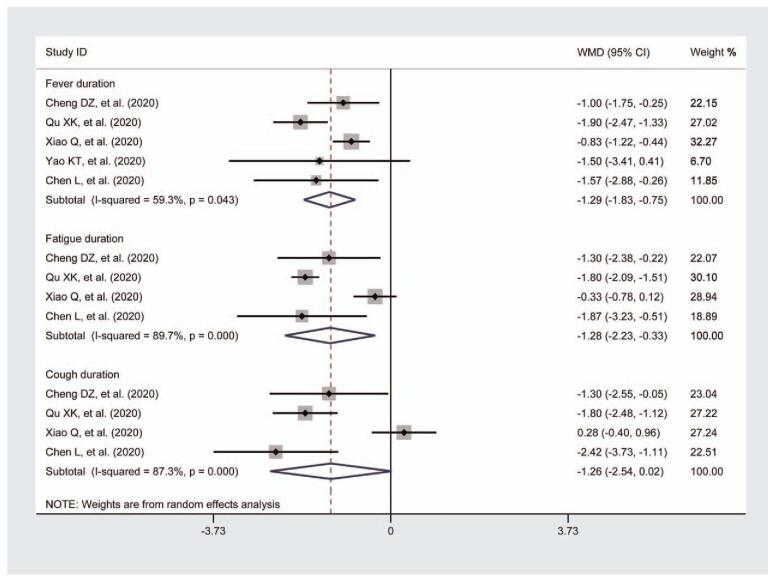
Meta-analysis for the comparison of duration of the main symptoms including fever, fatigue, and cough between the 2 groups. The meta-analysis results showed that compared to the Western medicine groups, the duration of each symptom in the trial groups did not show a significant difference. Heterogeneity was observed in the pooled results.

Five studies evaluated the effects of TCM on fever duration.^[22,23,25,28,29]^ A meta-analysis of 5 of these studies revealed that, compared to the Western medicine groups, the fever duration in the trial groups did not show a significant difference (n = 458; WMD = −1.29; 95% CIs = −1.83 to −0.75) (Fig. 4). Heterogeneity was observed in the pooled results (*I*-squared = 59.3%, *P* for heterogeneity = .043).

#### 3.6.2 Fatigue duration

In total, 4 studies provided data regarding the evaluation of fatigue duration in patients.^[22,23,25,29]^ The aggregated results suggested that therapy efficiency of fatigue duration was not significantly different between the 2 groups (n = 416; WMD = −1.28; 95% CIs = −2.23 to −0.33) (Fig. 4). A large heterogeneity was observed in the pooled outcomes (*I*-squared = 89.7%, *P* for heterogeneity = .000).

#### 3.6.3 Cough duration

Four studies were reported to evaluate the cough duration in patients.^[22,23,25,29]^ The meta-analysis revealed that there was no significant difference in the cough duration in the trial groups compared to the control groups (n = 416; WMD = −1.26; 95% CIs = −2.54 to 0.02) (Fig. 4). High heterogeneity was observed in the results (*I*-squared = 87.3%, *P* for heterogeneity = .000).

### 3.7 Analysis of publication bias

**Figure 5:**
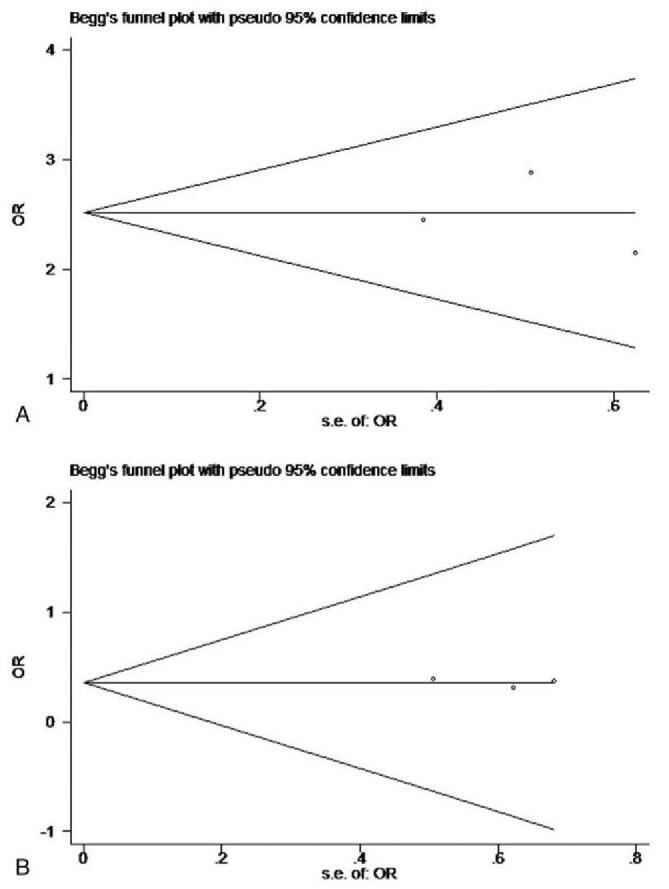
Results of publication bias involving evaluation index including total effective rate (A) and aggravation or hospitalization (B) using Begg methods. No evident publication bias was observed in this meta-analysis.

Begg funnel plot was used to assess the existence of publication bias. No evident publication bias was observed in our meta-analysis, for either the overall effective rate (Fig. 5A) or hospitalization (Fig. 5B) by visual inspection of the patterns of Begg funnel plot.

## 4 Discussion

### 4.1 Summary of evidence

After conducting a literature search of studies based on the eligibility criteria in both Chinese and English databases, only 8 studies were included to investigate the effectiveness of TCM in the treatment of COVID-19. Even though our included number of RCTs was small, these data are still essential and timely to guide doctors in the treatment of COVID-19. In this meta-analysis review, we performed the treatment effect of combining the utilization of TCM with Western medicine in COVID-19 patients. It was not surprising that the trial groups could perform better outcome measures because the TCM had been used for the treatment of influenza for a long time.^[15]^ At the detailed level, after the intervention of TCM into Western medicine in patients, the overall effective rate and disappearance rate (including fever, fatigue, cough, and sputum production), showed a significant improvement. However, no helpful effect was found in decreasing the duration of each symptom.

Additionally, the patient's age was also worthy of attention. The patients included in our 8 RCTs were aged between 33 and 75 years. In all included studies, Chinese medicine did not show any severe discomfort or abnormal effects during the whole treatment process (data not shown). Moreover, although there was no evidence to prove that the SARS-CoV-2 infections or morbidities of COVID-19 are related to the patient's age, the objective fact is that older adults have a relatively weaker immune system compared to young people, which means they probably are more susceptible to infections. In the present meta-analysis, we concluded that TCM is safe in older patients. Combining intervention with TCM and Western medicine can significantly improve aggravation or hospitalization.

Since patients could develop severe pulmonary disease and acute respiratory distress syndrome after being diagnosed with COVID-19, and autopsy histological examination showed substantial interstitial lymphocyte-dominated mononuclear inflammatory infiltrates, mortality could be attributed to the inflammatory responses.^[17]^ Therefore, anti-inflammatory agents probably could decrease the mortality induced by COVID-19.^[30]^ The main ingredients of Chinese medicines (including Shu-feng-jie-du, Lian-hua-qing-wen, and Jin-hua-qing-gan) are Forsythia (Lian-qiao), Lonicerae Japonicae Flos (Jin-yin-hua), Isatis root (Ban-lan-gen), and Radix glycyrrhizae (Gan-cao). Both honeysuckle and isatis root functions have anti-inflammatory and anti-bacterial effects in clinical use according to the Chinese medicine theory. Both components can enhance human immunity. In addition, Forsythia and Radix glycyrrhizae are normally used as clearing away of the “heat” and “fire” in the Chinese medicine theory, which means treating febrile diseases, acute upper respiratory tract infection, and cough yellow phlegm. As pharmaceutical therapies, Forsythia has many effects in inhibiting a variety of viruses, including influenza A virus, human cytomegalovirus, encephalitis B virus, and respiratory syncytial virus.^[31]^ Another Chinese medicine, Xue-bi-jing injection, can improve hemodynamics, reduce the levels of inflammatory factors such as TNF-a, inhibit excessive inflammatory response, and mitigate myocardial damage caused by sepsis.^[32]^ Thus, the utilization of TCM in managing COVID-19 is substantial.

### 4.2 Limitations of this review

The limitations of this review are considered as follows: First, the major limitation of this review is that we did not enclose a large number of studies. Because of this, the quality was highly restricted. Therefore, we believe that the significance of the conclusion may change if we enclose additional studies. Second, for the efficiency assessment, this review did not include as many outcome measures or inflammatory biomarkers to prove the validity of TCM. From the existing reports, in addition to fever, fatigue, dry cough, and sputum production, COVID-19 patients also presented with shortness of breath, myalgia, gastrointestinal symptoms, lymphopenia, prolonged prothrombin time, elevated C-reactive protein, etc.^[3,33,34]^ This meta-analysis focused only on the main outcome change (symptoms included fever, fatigue, dry cough, and sputum production) after intervention with TCM and Western medicine in COVID-19 patients. Third, the risk of bias of the included studies was unclear in general, which leads to a limitation in drawing a reliable conclusion on the effectiveness of TCM in the treatment of COVID-19. Fourth, the publications related to TCM are mostly published in Chinese because there were very few studies on RCTs related to it, and only Chinese patients were included. Moreover, clinical studies on COVID-19 are ongoing, and the results of the current analysis have not yet achieved the completeness of the evidence.

### 4.3 Implications for further research

Based on epidemiological data, COVID-19 showed a highly transmissible possibility as a novel coronavirus. Currently, there are no proven regimen drugs for the treatment of COVID-19. New therapeutic medicines and robust research are urgently needed. In light of the fact that TCM has been used widely in past epidemic diseases, TCM is still an essential part of the therapy of COVID-19. On the basis of our meta-analysis results, Chinese medicine showed a potential therapy function in treating COVID-19, significantly increasing the effective rate and improving the symptom disappearance rate.

Thus, considering several therapy stages (including prevention stage, mild stage, moderate stage, severe stage, and recovery stage) of this communicable disease, TCM could be applied as an alternative approach based on different compositions. For example, in the prevention stage, Radix astragali (Huang-qi), Radix glycyrrhizae (Gan-cao), Radix saposhnikoviae (Fang-feng), Rhizoma Atractylodis Macrocephalae (Bai-zhu), Lonicerae Japonicae Flos (Jin-yin-hua), and Fructus Forsythia (Lian-qiao) could be a good option for the COVID-19 high-risk population.^[35]^ As a review pointed out, after standardizing the terminology of the pattern identifications (PIs) and herbal formulae, there were 8 PIs and 23 herbal formulae for the mild stage, 11 PIs and 31 herbal formulae for the moderate stage, 8 PIs and 21 herbal formulae for the severe stage, and 6 PIs and 23 herbal formulae for the recovery stage in the Chinese guidelines.^[20]^ In light of the relation between TCM compositions and infectious-therapy stages, we are conducting a more comprehensive systematic review in the future to summarize the performance of TCM to COVID-19, which could be interesting.

Since this epidemic has not completely subsided, more large-sample clinical studies are still in progress, and the results of the current analysis have not yet achieved the completeness of the evidence. Therefore, this meta-analysis could only be used as a reference in the Chinese medicine treatment of COVID-19 in clinical settings. Based on our limited included studies, more clinical studies are still required to evaluate the efficacy and safety of TCM and to provide more clinical evidence. In the future, we are looking forward to further update and supplement the results of this system evaluation, and hopefully, integration of TCM into Western medicine could be an alternative option for the treatment of COVID-19.

## 5 Conclusions

Current evidence indicated that the therapy combined TCM with Western medicine had significant effects (in terms of overall effective rate, the effective rate of lung CT, and main clinical symptom reduction) in treating COVID-19 patients compared to Western medicine treatment alone. Supportive therapy using TCM revealed the potential adjunctive role of TCM in treating this devastating pandemic. However, high-quality clinical studies are still required to evaluate the efficacy and safety of TCM in the future treatment of COVID-19.

## Acknowledgments

The authors thank department members for their assistance with this study.

## Author contributions

**Conceptualization:** Feng Li, Lili Luan.

**Data curation:** Feng Li, Yongqing Jiang, Bei Yue.

**Formal analysis:** Feng Li, Bei Yue.

**Investigation:** Feng Li, Yongqing Jiang, Bei Yue.

**Methodology:** Feng Li, Bei Yue.

**Project administration:** Lili Luan.

**Resources:** Feng Li, Yongqing Jiang.

**Software:** Feng Li.

**Supervision:** Lili Luan.

**Validation:** Lili Luan.

**Writing – original draft:** Feng Li.

**Writing – review & editing:** Lili Luan.
